# Efficacy of venoarterial extracorporeal membrane oxygenation with and without intra-aortic balloon pump in adult cardiogenic shock

**DOI:** 10.3389/fcvm.2024.1431875

**Published:** 2024-09-06

**Authors:** Haiwang Wang, Chuanlong Li, Duo Li, Yuansen Chen, Wenli Li, Yanqing Liu, Yongnan Li, Haojun Fan, Shike Hou

**Affiliations:** ^1^ Institute of Disaster and Emergency Medicine, Tianjin University, Tianjin, China; ^2^ Wenzhou Safety (Emergency) Institute, Tianjin University, Wenzhou, China; ^3^ Laboratory of Extracorporeal Life Support, Lanzhou University Second Hospital, Lanzhou University, Lanzhou, China

**Keywords:** cardiogenic shock, venoarterial extracorporeal membrane oxygenation, intra-aortic balloon pump, survival, complications, meta-analysis

## Abstract

**Introduction:**

Intra-aortic balloon pump (IABP) is sometimes coupled with Venoarterial extracorporeal membrane oxygenation (VA-ECMO) to treat patients with cardiogenic shock. In this study, we attempted to evaluate the association of the IABP approach on survival and vascular complication rates in adults with cardiogenic shock undergoing VA-ECMO.

**Methods:**

We performed a systematic search of original studies on VA-ECMO with and without IABP in PubMed, EMBASE, and the Cochrane Library.

**Results:**

A total of 42 studies with 8,759 patients were included. The pooled in-hospital deaths of patients on VA-ECMO with and without IABP were 2,962/4,807 (61.61%) versus 2,666/3,952 (67.45%). VA-ECMO with IABP presents lower in-hospital mortality (risk ratio, 0.88; 95% CI, 0.86-0.91; *P* < 0.00001). In addition, IABP was associated with lower in-hospital mortality of patients with postcardiotomy cardiogenic shock and ischaemic heart disease. (risk ratio, 0.93; 95% CI, 0.87–0.98; *P* = 0.01; risk ratio, 0.85; 95% CI, 0.82–0.89; *P* < 0.00001). There was no significant difference in in-hospital morbidity in neurological, gastrointestinal, limb-related, bleeding, and infection complications between patients on VA-ECMO with and without IABP.

**Discussion:**

In these observational studies, concomitant use of IABP and VA-ECMO in adult patients with cardiogenic shock was associated with reduced in-hospital mortality.

**Systematic Review Registration:**

PROSPERO [CRD42017069259].

## Introduction

Cardiogenic shock (CS) is defined as a complex state of systemic hypoperfusion and tissue hypoxia due to a significant decrease in cardiac output (1, 2). The most common cause of CS is acute myocardial ischemia with left ventricular (LV) dysfunction (3). Despite significant advancements in revascularization strategies and heart failure pharmacotherapies, CS remains a major cause of morbidity and mortality (4–6), especially in-hospital mortality up to 50% (7).

Venoarterial extracorporeal membrane oxygenation (VA-ECMO) is a commonly used temporary mechanical circulatory support to maintain end-organ perfusion during the shock state (8), providing a crucial time window for cardiac recovery, switching to durable mechanical circulatory support, or heart transplant assessment (9). However, peripheral cannulation for VA-ECMO relies on retrograde aortic flow to perfuse vital organs (10), which can increase LV afterload (11, 12), often leading to decreased LV ejection and raised LV end-diastolic pressure (13, 14). The LV distention can lead to complications such as myocardial ischemia, delayed ventricular recovery, ventricular arrhythmias, pulmonary edema, thrombotic events, and multiorgan dysfunction (15–18). To prevent LV distension, intra-aortic balloon pump (IABP) counter-pulsation is sometimes used concomitantly with VA-ECMO in some centers. Theoretically, the role of IABP could reduce cardiac afterload and improve clinical outcomes (14). However, the actual benefits of VA-ECMO plus IABP in clinical is controversial in some recent studies (19–21). In 2018, we published a systematic review and meta-analysis of 29 retrospective cohort studies including 4,576 patients, which showed that the use of IABP for VA-ECMO patients on CS was associated with lower in-hospital mortality rates. Since then, the number of retrospective cohort studies on VA-ECMO plus IABP vs. VA-ECMO alone has substantially increased (22–26). Furthermore, there also some meta-analyses reported different results about the role of IABP during VA-ECMO (21, 27).

Moreover, the additional femoral arterial cannulation is associated with a risk of vascular complications including bleeding, distal limb ischemia, and infection (21, 28). Yang and colleagues reported that vascular complications are common and associated with lower survival in adult CS patients undergoing VA-ECMO support (29). In particular, the concomitant with IABP under VA-ECMO support was independent risk factor of vascular complications (29). Therefore, it is essential to assess the incidence of vascular complications in patients during VA-ECMO plus IABP support. However, there was limited positive comparison of the risk-benefit ratio in relevant complications between VA-ECMO plus IABP and VA-ECMO. To address this knowledge gap, we conducted an updated systematic review of state-of-the-art data concerning the use of IABP on clinical outcomes in patients treated with VA-ECMO for cardiogenic shock. It is important to note that we should not only pay attention to the survival benefits of IABP during VA-ECMO support but also the associated complications, including bleeding, and infection.

## Methods

### Data sources and search strategies

These data sources and search strategies were based on our previous report, registered in the International Prospective Register of Systematic Reviews (PROSPERO) [identifier (ID) CRD42017069259]. This systematic review was performed based on the Preferred Reporting Items for systematic reviews and Meta-Analyses (30). A comprehensive literature search was conducted on 31 May 2023 using PubMed, EMBASE, and the Cochrane library with the following MeSH, EMTREE, and free-text keywords: “extracorporeal membrane oxygenation”, “extracorporeal life support”, “intra-aortic balloon pumping”, “counterpulsation”, “left ventricular unloading or left ventricular unloading techniques”. The published date is between 13 June 2017 (finish date of the original report) and 31 May 2023 (finish date of the current update). There was not any restriction in this research. We included studies that: (1) all adults (≥18 years) patients receiving VA-ECMO with peripheral femoral-femoral or central cannulation (Supplementary Material 1); (2) compared patients with and without IABP under VA-ECMO support (Supplementary Materials 2, 3); (3) provided data on mortality in patients either 30-day or in-hospital (short-term mortality). The resulting citations were imported to EndNote V.X9(Thomson-Reuters; 2018, New York, USA) and duplicates were removed. All the titles and abstracts of each study were screened by 2 independent reviewers (HW and CL) to identify relevant studies. Then they reviewed the full text of all the relevant studies and extracted the data that met all inclusion criteria. Any discrepancies were resolved by consensus with a third independent reviewer (YL).

Consistent with our original systematic research (31), the primary outcome was in-hospital mortality. The secondary outcomes included neurological, limb-related, gastrointestinal complications, bleeding, and infection complications.

### Data synthesis and analysis

The extracted data were entered into Microsoft Excel (V.2019; Microsoft, USA) for further analysis. Statistical analysis was performed using RevMan 5.4 software (Cochrane Collaboration, Nordic Cochrane Centre, Copenhagen) and Stata (V.17 StataCorp). The continuous and binary variables were presented as mean difference or risk ratio (RR) with their 95% confidence intervals (CIs). The heterogeneity of the studies was evaluated using Cochrane Q tests or I^2^ values. If significant heterogeneity was present (I^2^ ≥ 50% or *p* < 0.1), pooled RR was used based on a random-effects model. Publication bias was evaluated using a funnel plot with 95% control limits if including more than 10 studies in Stata (V.17 StataCorp).

## Results

### General characteristics of the included studies

**Figure 1:**
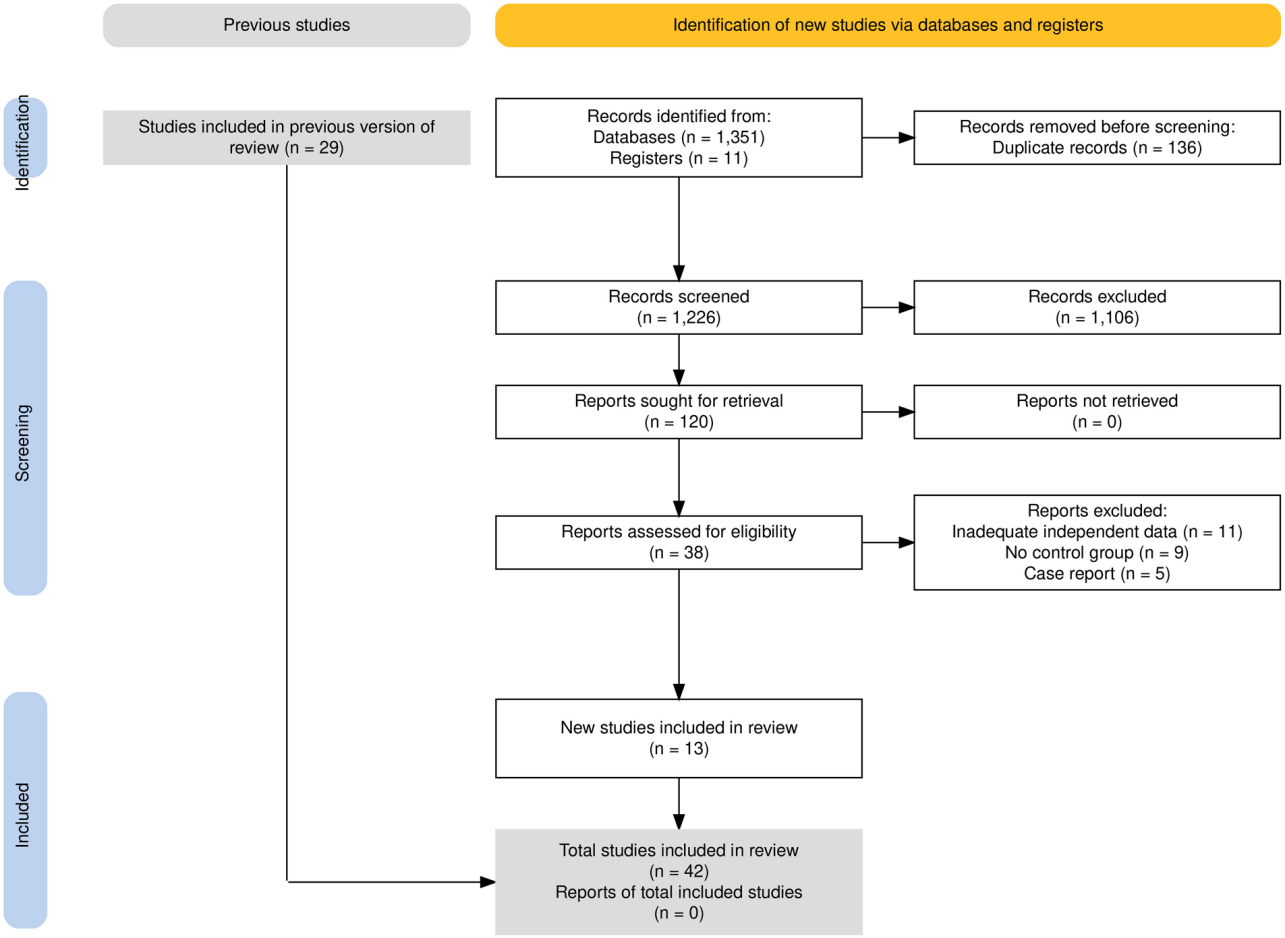
Flowchart of the study selection process.

The study selection process is outlined in Figure 1. A total of 1,360 records were obtained by searching the proposed database and 2 additional records were obtained by hand search of references. After deduplication and checking the abstract of searches, 38 full texts of records were acquired and independently reviewed. Finally, a total of 13 studies were included in the updated quantitative analysis (22–26, 32–39). All studies included were retrospective cohort studies. The characteristics of the newly included studies are summarized in Table 1 and the previous data before 2017 are shown in Supplementary Material Table S1.

**Table 1:** Characteristics of included studies from 2017 to 2023.

Study	Study type	Study period	Average years		Men, *n* (%)		Number of patients		Patient type	Peripheral ECMO, n(%)	Average time on ECMO		Survival to D/C,n(%)	Country/district
			ECMO	ECMO + IABP	ECMO	ECMO + IABP	ECMO	ECMO + IABP			ECMO	ECMO + IABP		
Arafat et al. (32)	RCS	01/2009–12/2020	62 (46.0–68.0)	60 (49–68.5)	20 (46.5)	24 (40.0)	43	60	PCS	17 (16.5)	88.5 (47.0–228.5)h	110.5 (68.2–172.3)h	40 (38.8)	Saudi Arabia
Barge-Caballero et al. (33)	RCS	01/2010–12/2015	50.9 ± 13.3	49.4 ± 12.7	73 (76.0)	55 (75.3)	96	73	IHD, PCS	161 (95.3)	9.5 days	10 days	NA	Spain
Bjornsdottir et al. (34)	RCS	01/2010–03/2018	62.0 ± 15.0	62.0 ± 15.0	80 (70.0)	80 (70.0)	114	114	PCS	149 (65.4)	5.3 (2.0–9.8) days	5.0 (2.9–8.9) days	89 (39.0)	European and Arabian
Brechot et al. (22)	RCS	01/2007–12/2012	53 (43–61)	52 (44–62)	44 (69.8)	50 (79.4)	63	63	IHD, MI	126 (100)	3 (2.0–6.0) days	4 (2–7) days	63 (50.0)	France
Brink et al. (35)	RCS	01/2015–12/2018	59 ± 7	59 ± 11	14 (78.0)		11	7	IHD	18 (100)	4.9 ± 2.8 days	4.5 ± 2.1 days	13 (72)	Netherlands
Char et al. (23)	RCS	01/2015–06/2020	58.0 (48.0–70.0)	59.5 (47.0–68.5)	83 (58.0)	47 (69.1)	143	68	IHD, MI	NA	4.0 (1.0–9.0) days	6.0 (3.0–10.5) days	96 (45.5)	USA
Chen et al. (36)	RCS	01/2005–12/2017	49.5 ± 14.1		112 (73.7)		75	77	PCS	152 (100)	4.8 days	6.6 days	73 (48)	China
Djordjevic et al. (37)	RCS	03/2006–03/2017	66 (55,73)	66 (55,73)	24 (56.0)	122 (79.0)	43	129	PCS	117 (68.0)	44 h	68 h	45 (26)	Germany
Kida et al. (25)	RCS	01/1998–12/2014	70.84 ± 11.0	66.35 ± 12.00	406 (78.5)	367 (80.1)	60	459	IHD	NA	NA	NA	229 (44.1)	Japan
Kuroki et al. (38)	RCS	01/2010–12/2017	64.1 ± 15.3	63.1 ± 13.6	128 (74.0)	635 (83.0)	173	762	IHD, MI	184 (100)	NA	NA	326 (35)	Japan
Monaco et al. (39)	RCS	02/2013–09/2019	67 (60–73)	66 (59–71)	69 (90.7)	43 (95.6)	76	45	IHD	121 (100)	23.5 h	24.0 h	117 (96.7)	Italy
Nishi et al. (26)	RCS	04/2012–03/2018	69 (60, 78)	69 (61, 77)	664 (78.5)	652 (77.1)	846	846	IHD	NA	NA	NA	1,581 (93.4)	Japan
Tepper et al. (24)	RCS	02/2010–06/2016	50.5 ± 17.7	57.2 ± 10.6	14 (47.0)	18 (60.0)	30	30	PCS	0 (0)	NA	NA	19 (31.7)	USA

ECMO, extracorporeal membrane oxygenation; IABP, intra-aortic balloon pump; D/C, hospital discharge; RCS, retrospective cohort study; PCS, postcardiotomy cardiogenic shock; IHD, ischaemic heart disease; MI, myocarditis; NA, not available.

All studies included were assessed with the Newvastle-Ottawa Scale: 9 of them were considered as high quality; 29 were identified as moderate; 4 were considered as low quality ( Supplementary Material Table S2 ).

### Participants characteristics

A total of 8,759 patients were included (3,952 ECMO alone vs. 4,807 ECMO plus IABP) and the baseline demographics were presented in Table 1. The mean age of the patients was 59.8 years; 71.4% were men. A total of 5 studies adopted peripheral VA-ECMO and another 9 studies used both central and peripheral cannulation (Supplementary Material).

According to the etiology of the CS, the enrolled patients were divided into three types, including postcardiotomy cardiogenic shock (PCS), ischaemic heart disease (IHD), and myocarditis. A total of 17 studies reported on PCS of patients and the survival rate was about 35.4% (1,006/2,840). Besides, another 13 studies reported that patients due to IHD showed a survival rate of 25.6% (724/2,824). Two studies that reported on myocarditis demonstrated a survival rate of 70.8% (17/24).

### In-hospital mortality rate

**Figure 2:**
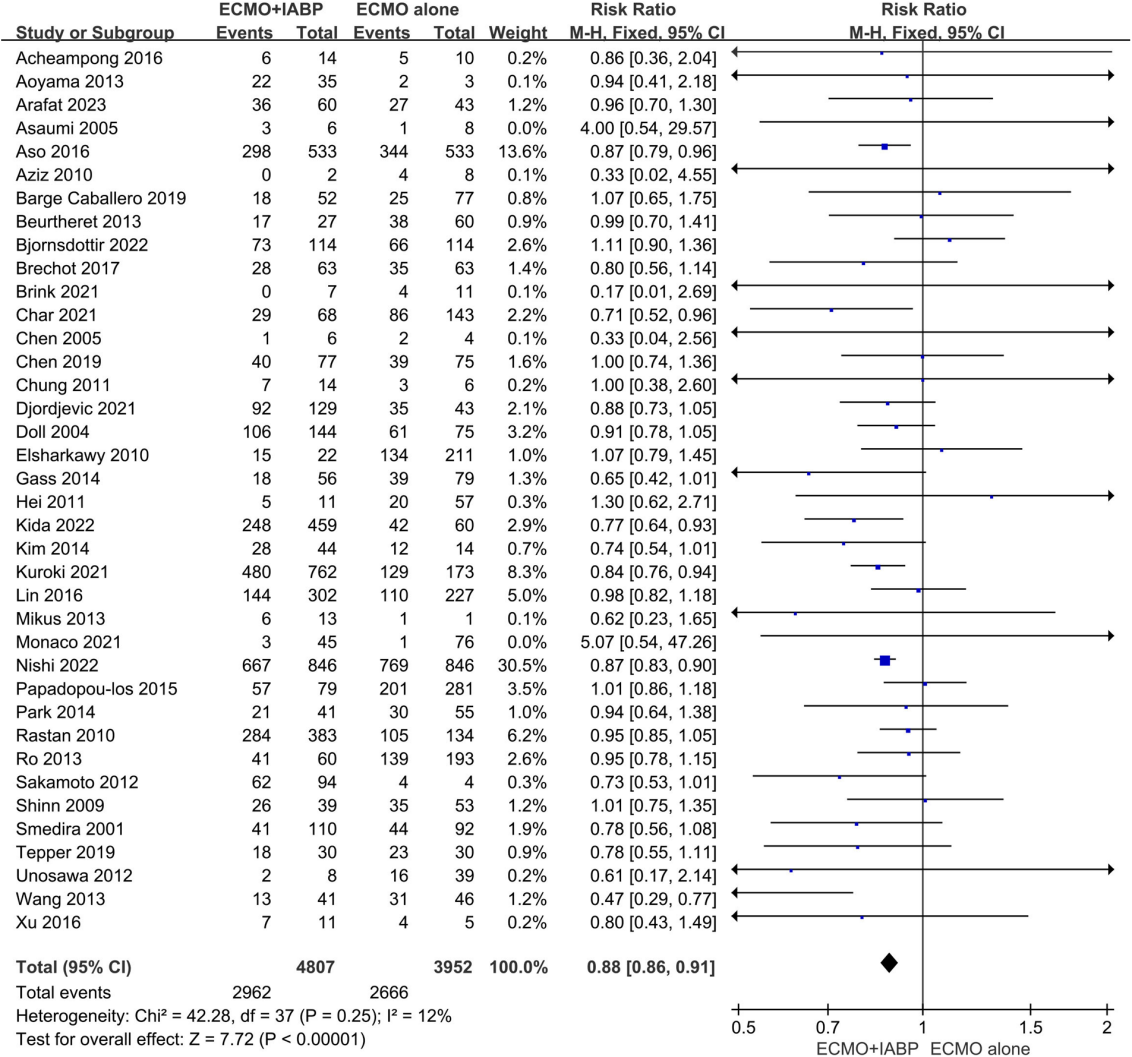
Forest plot of in-hospital mortality rates in patients treated with venoarterial ECMO with IABP vs. venoarterial ECMO. CI: confidence interval; ECMO: extracorporeal membrane oxygenation; IABP: intra-aortic balloon pump; M-H: Mantel–Haenszel.

Overall, in-hospital mortality was significantly lower in patients combined with IABP than VA-ECMO alone (RR 0.88; 95% CI 0.86–0.91, I^2 ^= 12%; *P *< 0.00001, Figure 2). The funnel plot was stacked and all points were under the funnel after 1 outlying study was removed, indicating that there was no obvious publication bias after adjustment (Supplementary Material Figures S1A and B). Also, with the study removed, there was no obvious difference (RR 0.88; 95% CI 0.85–0.91, I^2 ^= 10%; *P *< 0.00001, Supplementary Material Figure S3A). Meanwhile, avoiding the data overlap between included studies, the larger one from Japan by Nishi et al (26) was reserved in the meta-analysis (Supplementary Material Figure S3B). The result was also similar with a previous cumulative in-hospital mortality rate (RR 0.90; 95% CI 0.86–0.93, I^2 ^= 15%; *P *< 0.00001). In addition, to ensure the accuracy of the results, we calculated repeatedly without the study (26), and the result has no difference (RR 0.89; 95% CI 0.85–0.93, I^2 ^= 13%; *P *< 0.0001, Supplementary Material Figure S3C). The sensitivity analysis was checked on STATA software and the result was stable (Supplementary Material Figure S2). Moreover, among the 13 updated studies, the in-hospital mortality exhibited a similar trend as previous data (RR 0.87; 95% CI 0.84–0.90, I2 = 19%; *P* < 0.00001, Supplementary Material Figure S5).

**Figure 3:**
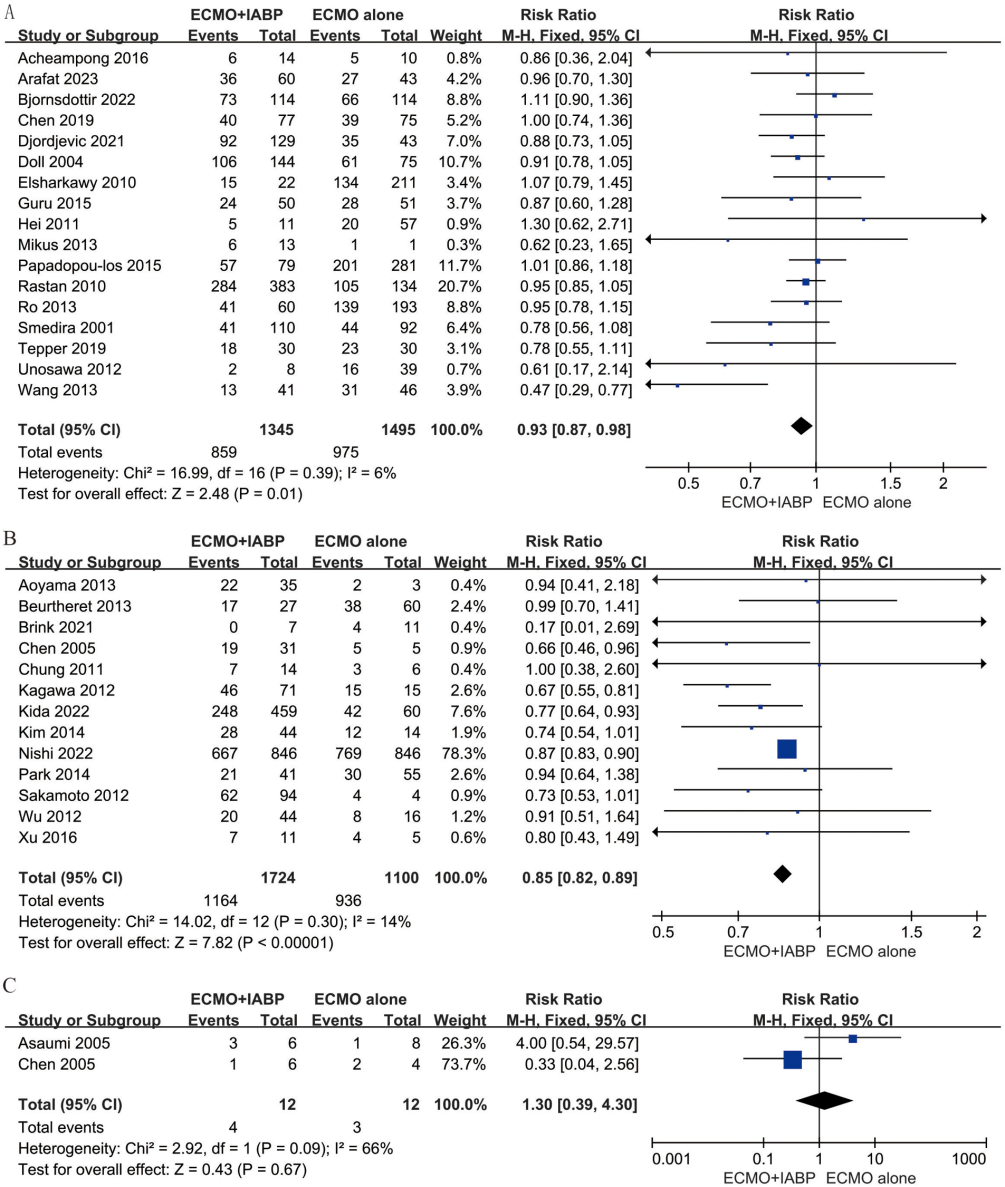
Forest plot of in-hospital mortality rates in patients with postcardiotomy **(A)**, ischaemic heart disease **(B)**, and myocarditis **(C)** under venoarterial ECMO with IABP vs. venoarterial ECMO. CI: confidence interval; ECMO: extracorporeal membrane oxygenation; IABP: intra-aortic balloon pump; M-H: Mantel–Haenszel.

Subgroup analysis stratified by etiology of CS presented that combined with IABP has an improvement in survival rate by PCS (RR 0.93; 95% CI 0.87–0.98, I^2 ^= 6%; *P *= 0.01, Figure 3A) and IHD (RR 0.85; 95% CI 0.82–0.89, I^2 ^= 14%; *P *< 0.00001, Figure 3B). In-hospital mortality was comparable between VA-ECMO combined with IABP and ECMO alone when the primary diagnosis was myocarditis (RR 1.30; 95% CI 0.39–4.30, I^2 ^= 66%; *P *= 0.67, Figure 3C).

### Secondary outcomes

**Figure 4:**
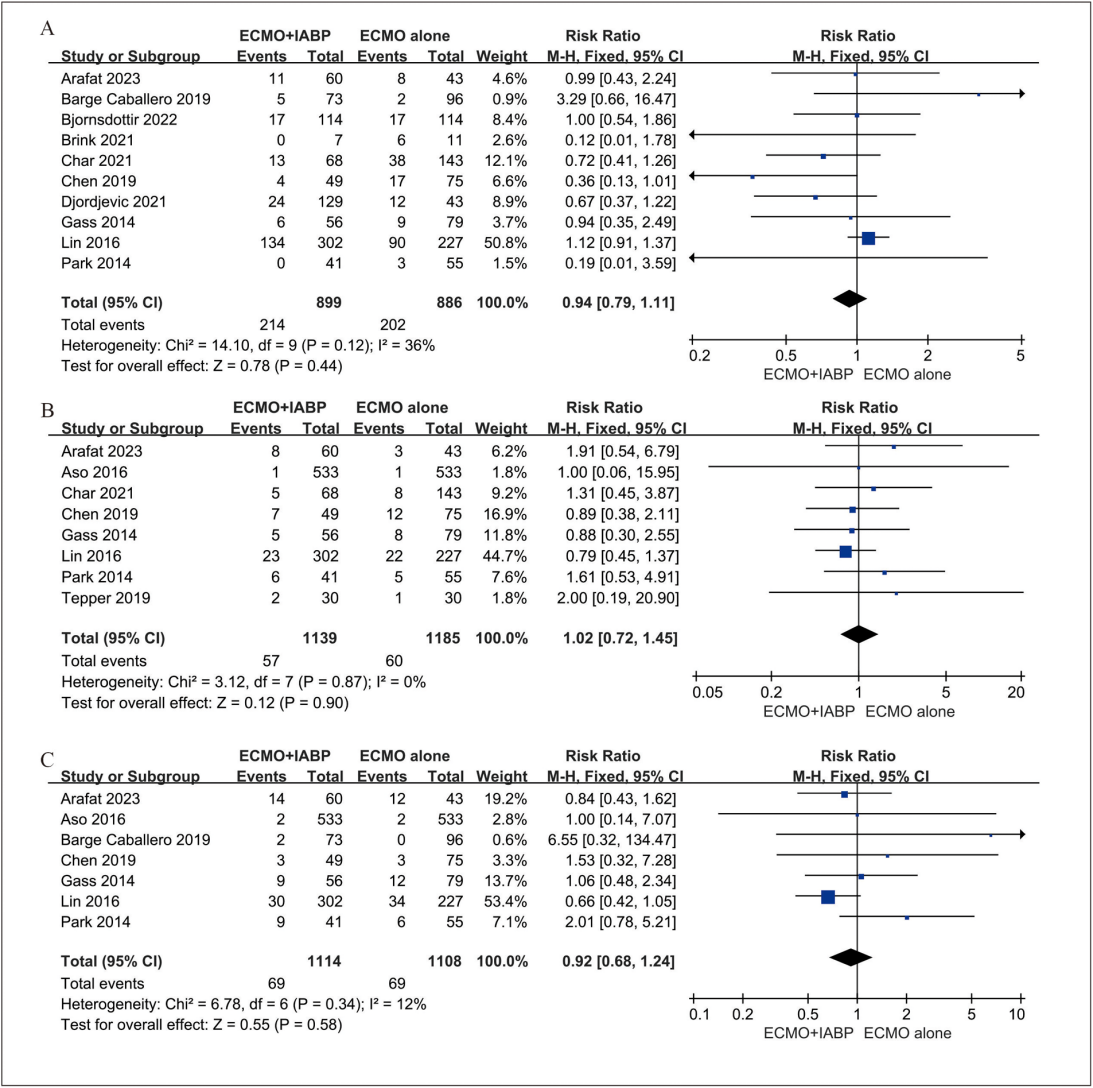
Forest plot of neurological **(A)**, limb-related **(B)**, and gastrointestinal **(C)** complications between venoarterial ECMO with IABP vs. venoarterial ECMO. CI: confidence interval; ECMO: extracorporeal membrane oxygenation; IABP: intra-aortic balloon pump; M-H: Mantel–Haenszel.

**Figure 5:**
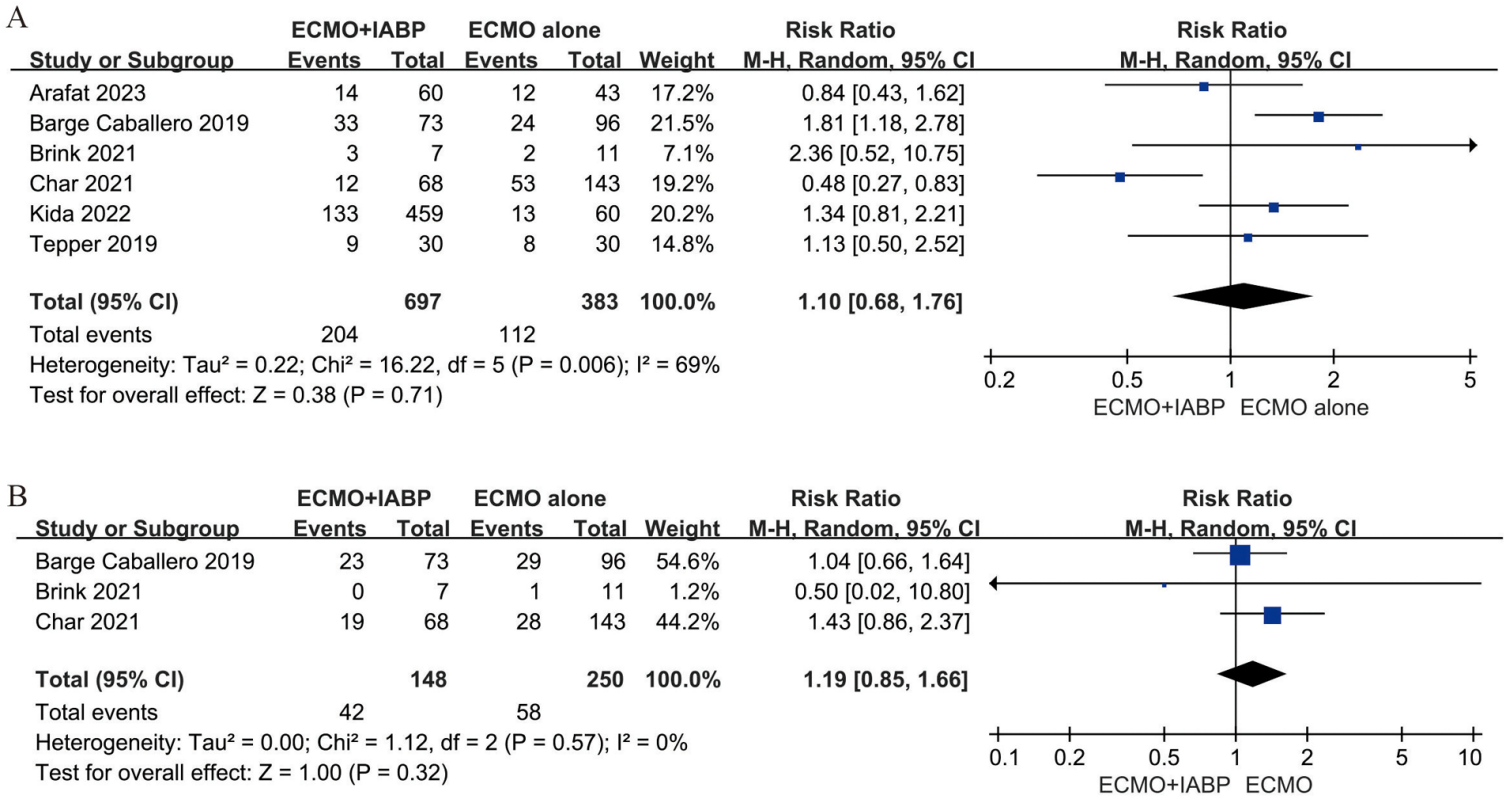
Forest plot of bleeding **(A)**, and infection **(B)** complications between venoarterial ECMO with IABP vs. venoarterial ECMO. CI: confidence interval; ECMO: extracorporeal membrane oxygenation; IABP: intra-aortic balloon pump; M-H: Mantel–Haenszel.

Four studies (23, 35–37) included 525 patients for neurological, four studies (23, 24, 32, 36) included 498 patients for limb-related and three studies (32, 33, 36) included 396 patients for gastrointestinal complications. In addition, six studies (23–25, 32, 33, 35) involved data for bleeding, stroke (32–34) and infection (23, 33, 35). The rate of neurological (RR 0.94; 95% CI 0.79–1.11, I^2 ^= 36%; *P *= 0.44, Figure 4A), limb-related (RR 1.02; 95% CI 0.72–1.45, I^2 ^= 0%; *P *= 0.90, Figure 4B), gastrointestinal (RR 0.92; 95% CI 0.68–1.24, I^2 ^= 12%; *P *= 0.58, Figure 4C), bleeding (RR 1.10; 95% CI 0.68–1.76, I^2 ^= 69%; *P *= 0.71, Figure 5A) and infection (RR 1.19; 95% CI 0.85–1.66, I^2 ^= 0%; *P *= 0.32, Figure 5B) were similar between patients treated with VA-ECMO with vs. without IABP.

## Discussion

This systematic review and meta-analysis displayed an updated use of IABP during VA-ECMO for cardiogenic shock using a large combined cohort. In comparison with the previous report in 2018, we included 42 studies, including 13 updated studies (29 studies were included in the previous report). Our updated meta-analysis demonstrated that patients treated with VA-ECMO plus IABP had less in-hospital mortality compared with similar patients in whom IABP was not used. The primary outcome was consistent with our previous research (31). Although restricted by the studies are retrospective cohort studies, our present meta-analysis update supports the use of IABP in appropriate patients with cardiogenic shock in whom VA-ECMO was used.

The patients with acute cardiovascular diseases, CS is a leading cause of mortality and morbidity in clinical. The most common causes of CS are postcardiotomy cardiogenic shock (PCS), ischaemic heart disease (IHD), and myocarditis. PCS remains a clinical challenge and occurs in 3%–5% of contemporary cardiac operations with mortality rates of 50%–80% (40–42). The use of VA-ECMO for adult PCS has increased, with a survival rate of 16%–42% (43, 44). Furthermore, Samsky et al. reported that the most common etiology of CS is IHD because of the occlusion of the epicardial coronary artery, leading to regional cardiac myocyte ischemia (1). In addition to some medical therapies, mechanical circulatory support has been proposed for the treatment of ventricular failure due to IHD in cardiogenic shock (45). In our updated meta-analysis, we found that IABP plays an important role in reducing the mortality rate of CS patients with the causes of PCS and IHD. Myocarditis is defined as inflammation of the heart muscle caused by viral, rickettsial, bacterial, or protozoal infections or drug toxicity (46). In clinical practice, patients with fulminant myocarditis often present with cardiogenic shock due to a severe left ventricular dysfunction. Some studies have shown that mechanical circulatory support is effective in patients with cardiogenic shock secondary to fulminant myocarditis (47, 48). However, there was no significant difference in patients with myocarditis in cardiogenic shock between VA-ECMO combined with IABP and ECMO alone in this study. The primary reason for the observed variable benefits between myocarditis and conditions such as IHD or post-cardiotomy is likely due to the limited number of patients enrolled in the study, which can lead to unreliable or inconclusive results.

Many factors should be considered when deciding whether to add the IABP on patients under VA-ECMO support, due to the potential complications (49). We also focused on the differences in complications due to IABP implantation, including neurological, limb-related, gastrointestinal, bleeding, and infection complications in this updated research. Mateen et al. reported neurological events that occurred in 42 patients out of 87 adults who were treated with ECMO, including subarachnoid hemorrhage, ischemic infarctions, etc (50). In addition, bleeding and thrombosis are the two most common complications between patients under VA-ECMO support (51). Chung et al. queried the Extracorporeal Life Support Organization (ELSO) database and reported that bleeding events are twice as common as thrombotic events, with a significant influence on survival (52). Furthermore, infection is also a severe complication during mechanical circulation support, resulting in fever and organ dysfunction.

As shown in Figures 4, 5, IABP did not demonstrate a complication benefit and saw modestly increased odds of limb-related, bleeding, stroke, and infection. It was worrying because the slight increase in the IABP group may be a direct result of its insertion. Several reasons may support the observation of a higher complication rate with IABP use. First, it has been demonstrated that VA-ECMO use alone also with higher complicated rates, including bleeding, limb-related ischemic, and stroke (53). The insertion of the second device through vascular further increased the likelihood of relevant complications. Second, the indicators of illness severity during VA-ECMO support. The need for LV unloading usually depends on the complex heart dysfunction during VA-ECMO support. Therefore, the rapid deterioration of the disease is also a cause of relevant complications. Third, the second device increased the length of stay in the hospital. Because of the usefulness of IABP during VA-ECMO and therefore a longer follow-up, they were also susceptible to complications.

To data, bleeding remains the most frequent complication in patients with VA-ECMO and is associated with significant morbidity (54). In some clinical observation studies of patients supported with ECMO including adults (55, 56) and children (57). Almost all patients suffered from acquired von Willebrand syndrome (AvWS), which can contribute to bleeding tendencies due to loss of the high molecular weight multimers of von Willebrand factor (vWF). Vincent et al. reported the association between the endothelial release of new vWF and vascular pulsatility (58). The mechanism of action of IABP allows for the delivery of pulsatile flow to the aorta, which is of significance in patients undergoing continuous flow VA-ECMO support. In addition, in recent report (18), the authors compared the effect between IABP and percutaneous ventricular assist device (pVAD) as mechanical unloading on VA-ECMO. It is of note that patients receiving VA-ECMO plus IABP exhibited a lower incidence of bleeding complications, particularly in hemorrhagic stroke (1.9% vs. 4.1%) and gastrointestinal bleeding (3.9% vs. 8.1%). These findings are particularly noteworthy given that gastrointestinal bleeding is considered a severe complication of AvWS patients under continuous flow devices support (59). In this study, there is no significant difference in gastrointestinal bleeding between VA-ECMO plus IABP and VA-ECMO alone (Figure 4C). It is probable that the sample size is insufficient to permit the detection of differences between the two groups. Further researches are needed in this area.

In a word, the decision to insert an LV unloading device is complex. The clinicians should carefully balance the benefits against the potentially higher complication rate. Besides, the relevant complications might be associated with anticoagulant strategies or aseptic operations rather than the difference in treatments. Finally, given the lower mortality compared VA-ECMO plus IABP with VA-ECMO alone and the signal for a slightly higher complication rate with IABP, a randomized trial of VA-ECMO plus IABP is urgently needed to improve the LV unloading strategy in the future.

### Limitations

Several limitations should be considered in the process of the updated meta-analysis. Firstly, due to only retrospective cohort studies included, selection bias was inevitable in this report. The net effect of IABP on patients treated with VA-ECMO is difficult to ascertain. Secondly, all the patients had different baselines, with different etiology of CS, and different levels of lactic acid, which may affect the outcomes. Finally, the current findings have a strong inclination to patients with PCS and IHD, the results may not be appropriate for other patients with other etiologies of CS.

## Conclusions

This updated meta-analysis also demonstrated that using IABP on patients treated with VA-ECMO for CS was associated with a decreased in-hospital mortality rate. Meanwhile, IABP not only demonstrated a complication benefit but also modestly increased the odds of limb-related, bleeding, stroke, and infection. So, clinicians need to consider the complexity of complications when deciding to use IABP during VA-ECMO support.

## Data Availability

The original contributions presented in the study are included in the article/Supplementary Material, further inquiries can be directed to the corresponding authors.
