# Supplementary material for: Efficacy of venoarterial extracorporeal membrane oxygenation with and without intra-aortic balloon pump in adult cardiogenic shock
Source: Front Cardiovasc Med. 2024 Sep 6;11:1431875. doi: 10.3389/fcvm.2024.1431875 (PMC11412878; doi:10.3389/fcvm.2024.1431875)
Supplement: Supplementary file 1 [file Datasheet1.pdf]

Supplementary Materials

Supplementary Figure 1: Funnel plot of in-hospital mortality in the patients treated with VA-ECMO plus IABP versus VA-ECMO. Except for Monaco 2021 (A). All studies spreading evenly on both sides of the average indicated no obvious publication bias (B). VA-ECMO = venoarterial extracorporeal membrane oxygenation, IABP = intra-aortic balloon pump.

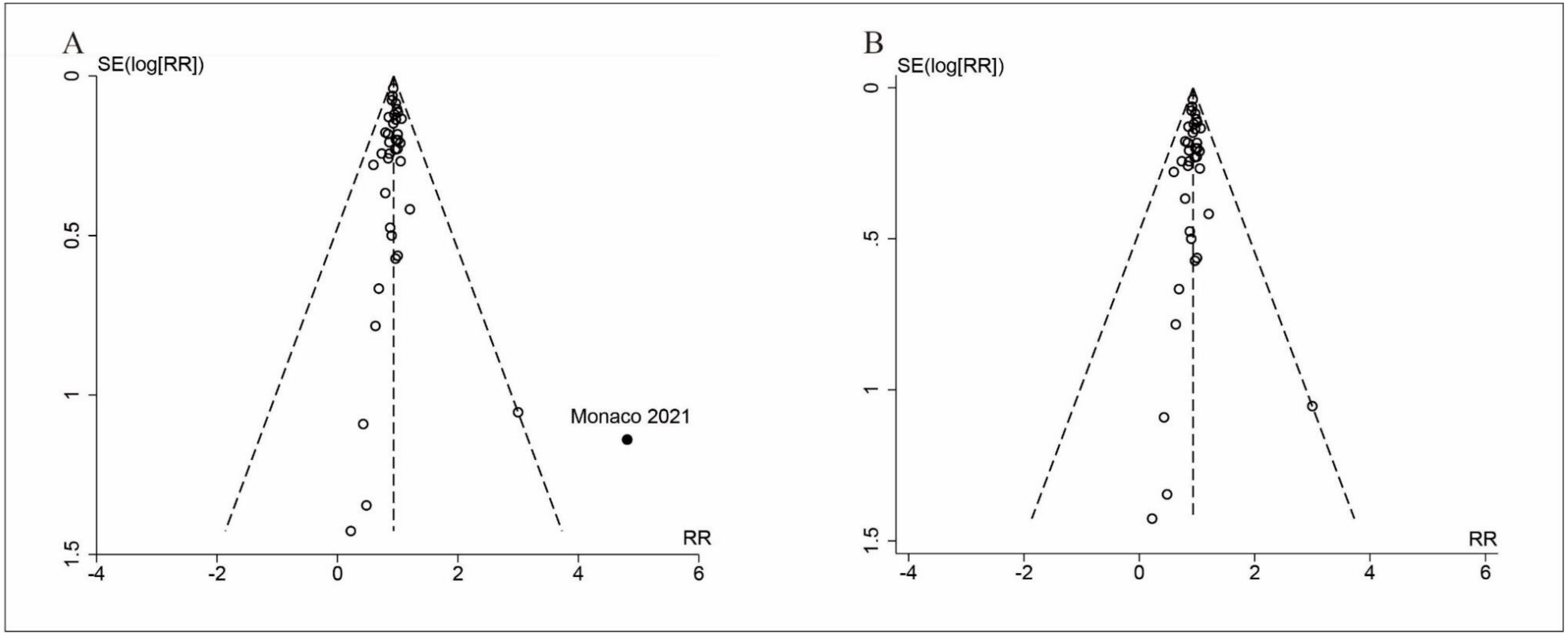

Supplementary Figure 2: The sensitivity was tested in STATA and the results were stable.

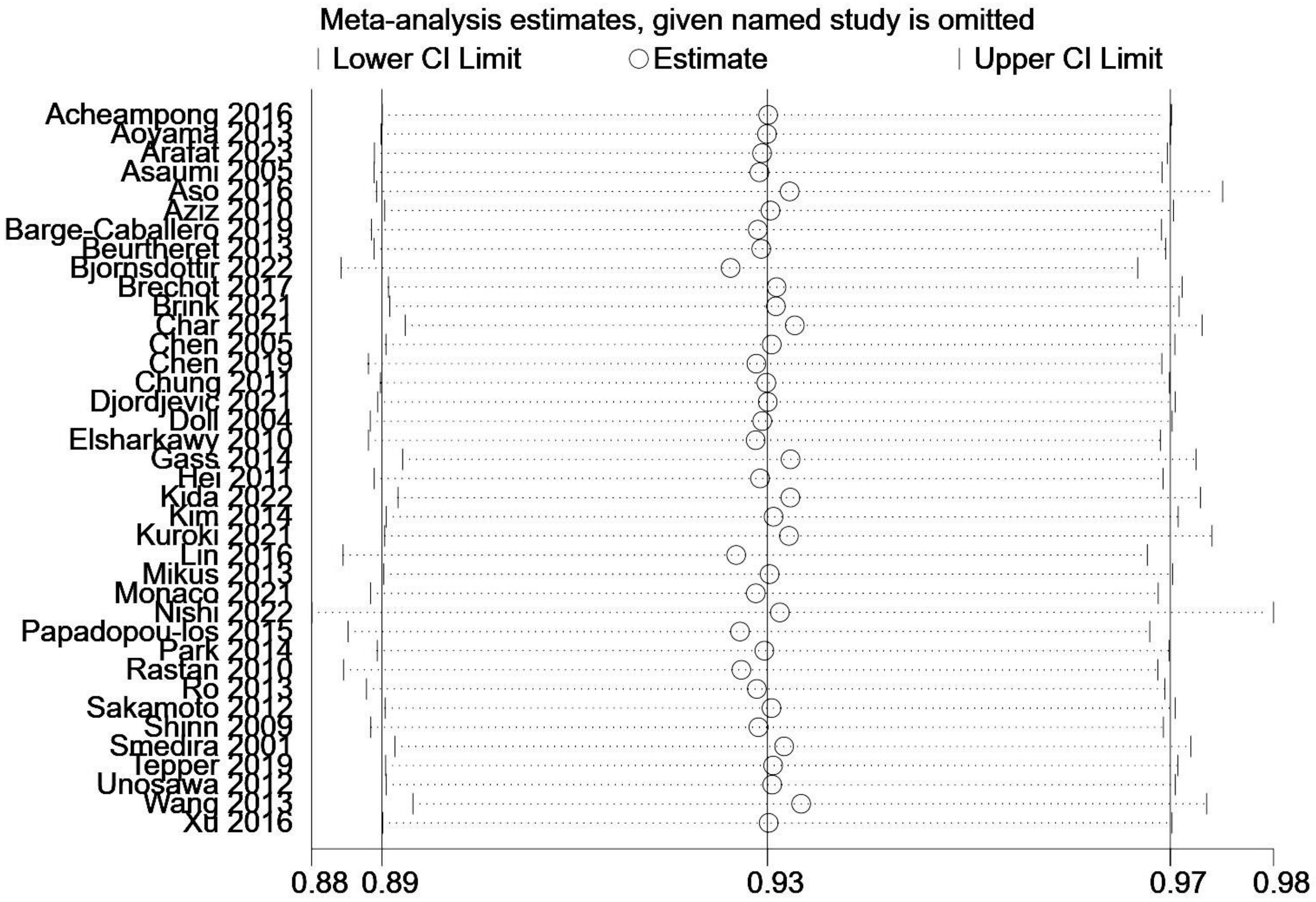

Supplementary Figure 3: Forest plot of in-hospital mortality in patients treated with extracorporeal cardiopulmonary resuscitation between venoarterial ECMO plus IABP and venoarterial ECMO. CI: confidence interval; ECMO: extracorporeal membrane oxygenation; IABP: intra-aortic balloon pump; M-H: Mantel–Haenszel.

	ECMO+IABP		ECMO alone			Risk Ratio
Study or Subgroup	Events	Total	Events	Total	Weight	M-H, Fixed, 95% CI
Chen 2005	19	31	5	5	12.5%	0.66 [0.46, 0.96]
Guru 2015	24	50	28	51	37.4%	0.87 [0.60, 1.28]
Kagawa 2012	46	71	15	15	34.2%	0.67 [0.55, 0.81]
Wu 2012	20	44	8	16	15.8%	0.91 [0.51, 1.64]
**Total (95% CI)**		**196**		**87**	**100.0%**	**0.78 [0.64, 0.95]**
Total events	109		56			

Heterogeneity: Chi² = 4.00, df = 3 (P = 0.26); I² = 25%
Test for overall effect: Z = 2.46 (P = 0.01)

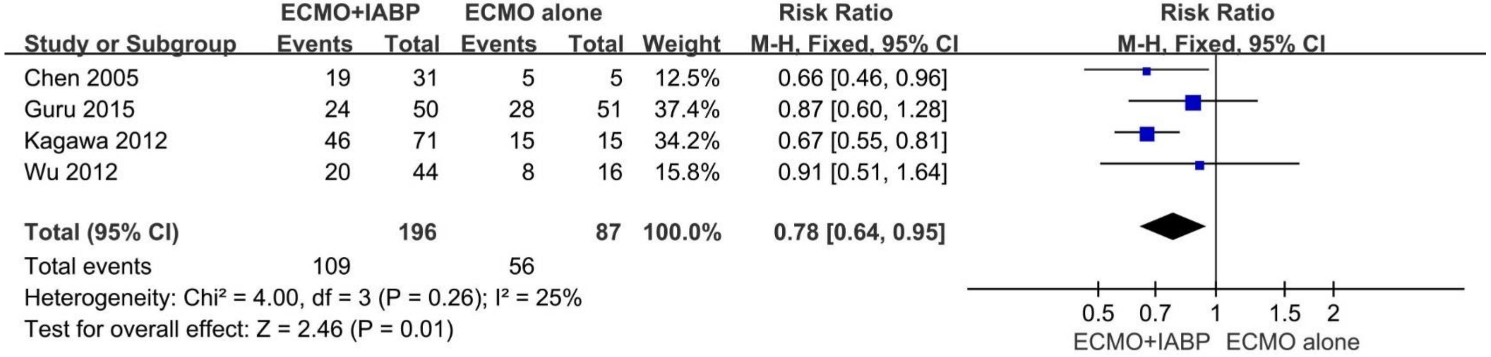

Supplementary Figure 4: Forest plot of in-hospital mortality in patients treated with venoarterial ECMO plus IABP and venoarterial ECMO. Removing Monaco 2021 (A), removing 7 studies from Japan (B), and removing Nishi 2022 (C).

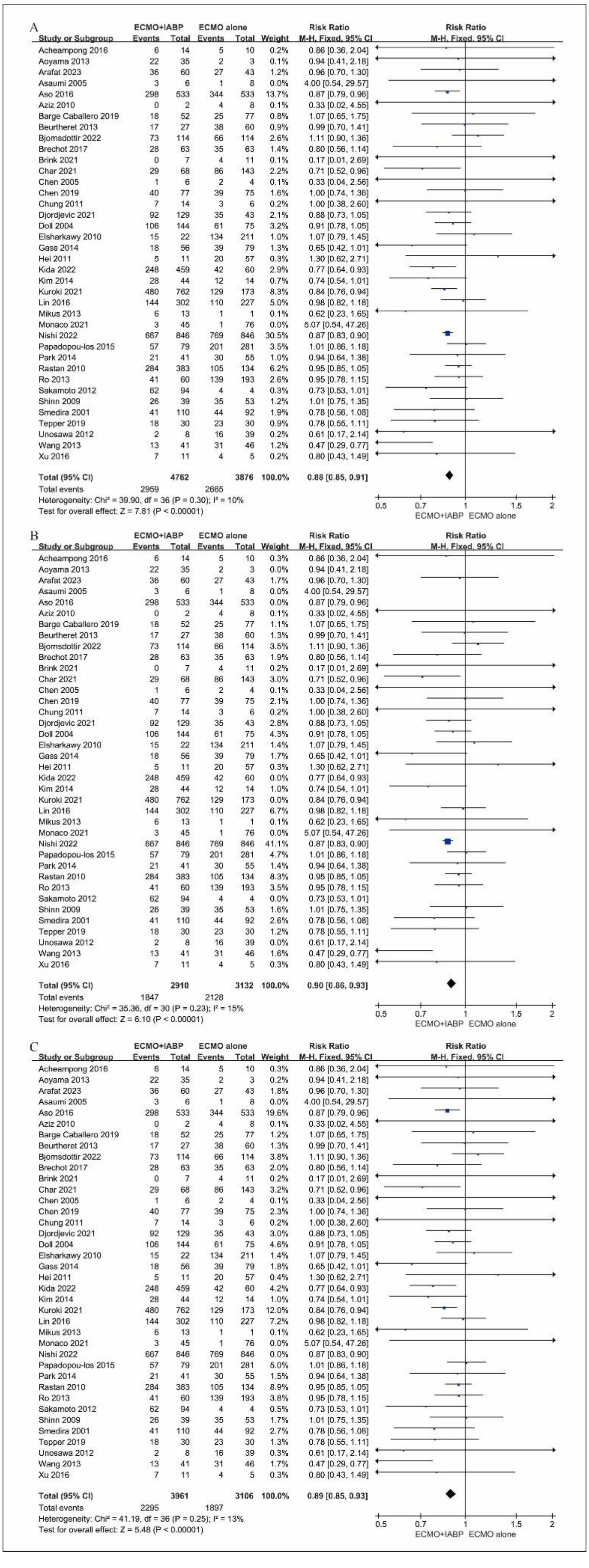
A
ECMO+IABP ECMO alone Risk Ratio Risk Ratio
Study or Subgroup Events Total Events Total Weight M-H, Fixed, 95% CI M-H, Fixed, 95% CI
Acheampong 2016 6 14 5 10 0.2% 0.86 [0.36, 2.04]
Aoyama 2013 22 35 2 3 0.1% 0.94 [0.41, 2.18]
Arafat 2023 36 60 27 43 1.2% 0.96 [0.70, 1.30]
Asaumi 2005 3 6 1 8 0.0% 4.00 [0.54, 29.57]
Aso 2016 298 533 344 533 13.7% 0.87 [0.79, 0.96]
Aziz 2010 0 2 4 8 0.1% 0.33 [0.02, 4.55]
Barge Caballero 2019 18 52 25 77 0.8% 1.07 [0.65, 1.75]
Beurtheret 2013 17 27 38 60 0.9% 0.99 [0.70, 1.41]
Bjomsdottir 2022 73 114 66 114 2.6% 1.11 [0.90, 1.36]
Brechot 2017 28 63 35 63 1.4% 0.80 [0.56, 1.14]
Brink 2021 0 7 4 11 0.1% 0.17 [0.01, 2.69]
Char 2021 29 68 86 143 2.2% 0.71 [0.52, 0.96]
Chen 2005 1 6 2 4 0.1% 0.33 [0.04, 2.56]
Chen 2019 40 77 39 75 1.6% 1.00 [0.74, 1.36]
Chung 2011 7 14 3 6 0.2% 1.00 [0.38, 2.60]
Djordjevic 2021 92 129 35 43 2.1% 0.88 [0.73, 1.05]
Doll 2004 106 144 61 75 3.2% 0.91 [0.78, 1.05]
Elsharkawy 2010 15 22 134 211 1.0% 1.07 [0.79, 1.45]
Gass 2014 18 56 39 79 1.3% 0.65 [0.42, 1.01]
Hei 2011 5 11 20 57 0.3% 1.30 [0.62, 2.71]
Kida 2022 248 459 42 60 2.9% 0.77 [0.64, 0.93]
Kim 2014 28 44 12 14 0.7% 0.74 [0.54, 1.01]
Kuroki 2021 480 762 129 173 8.3% 0.84 [0.76, 0.94]
Lin 2016 144 302 110 227 5.0% 0.98 [0.82, 1.18]
Mikus 2013 6 13 1 1 0.1% 0.62 [0.23, 1.65]
Monaco 2021 3 45 1 76 0.0% 5.07 [0.54, 47.26]
Nishi 2022 667 846 769 846 30.5% 0.87 [0.83, 0.90]
Papadopou-los 2015 57 79 201 281 3.5% 1.01 [0.86, 1.18]
Park 2014 21 41 30 55 1.0% 0.94 [0.64, 1.38]
Rastan 2010 284 383 105 134 6.2% 0.95 [0.85, 1.05]
Ro 2013 41 60 139 193 2.6% 0.95 [0.78, 1.15]
Sakamoto 2012 62 94 4 4 0.3% 0.73 [0.53, 1.01]
Shinn 2009 26 39 35 53 1.2% 1.01 [0.75, 1.35]
Smedira 2001 41 110 44 92 1.9% 0.78 [0.56, 1.08]
Tepper 2019 18 30 23 30 0.9% 0.78 [0.55, 1.11]
Unosawa 2012 2 8 16 39 0.2% 0.61 [0.17, 2.14]
Wang 2013 13 41 31 46 1.2% 0.47 [0.29, 0.77]
Xu 2016 7 11 4 5 0.2% 0.80 [0.43, 1.49]
Total (95% CI) 4762 3876 100.0% 0.88 [0.85, 0.91]
Total events 2959 2665
Heterogeneity: Chi² = 39.90, df = 36 (P = 0.30); I² = 10%
Test for overall effect: Z = 7.81 (P < 0.00001)
0.5 0.7 1 1.5 2
ECMO+IABP ECMO alone
B
ECMO+IABP ECMO alone Risk Ratio Risk Ratio
Study or Subgroup Events Total Events Total Weight M-H, Fixed, 95% CI M-H, Fixed, 95% CI
Acheampong 2016 6 14 5 10 0.3% 0.86 [0.36, 2.04]
Aoyama 2013 22 35 2 3 0.0% 0.94 [0.41, 2.18]
Arafat 2023 36 60 27 43 1.7% 0.96 [0.70, 1.30]
Asaumi 2005 3 6 1 8 0.0% 4.00 [0.54, 29.57]
Aso 2016 298 533 344 533 0.0% 0.87 [0.79, 0.96]
Aziz 2010 0 2 4 8 0.1% 0.33 [0.02, 4.55]
Barge Caballero 2019 18 52 25 77 1.1% 1.07 [0.65, 1.75]
Beurtheret 2013 17 27 38 60 1.3% 0.99 [0.70, 1.41]
Bjomsdottir 2022 73 114 66 114 3.5% 1.11 [0.90, 1.36]
Brechot 2017 28 63 35 63 1.9% 0.80 [0.56, 1.14]
Brink 2021 0 7 4 11 0.2% 0.17 [0.01, 2.69]
Char 2021 29 68 86 143 3.0% 0.71 [0.52, 0.96]
Chen 2005 1 6 2 4 0.1% 0.33 [0.04, 2.56]
Chen 2019 40 77 39 75 2.1% 1.00 [0.74, 1.36]
Chung 2011 7 14 3 6 0.2% 1.00 [0.38, 2.60]
Djordjevic 2021 92 129 35 43 2.8% 0.88 [0.73, 1.05]
Doll 2004 106 144 61 75 4.3% 0.91 [0.78, 1.05]
Elsharkawy 2010 15 22 134 211 1.4% 1.07 [0.79, 1.45]
Gass 2014 18 56 39 79 1.7% 0.65 [0.42, 1.01]
Hei 2011 5 11 20 57 0.3% 1.30 [0.62, 2.71]
Kida 2022 248 459 42 60 0.0% 0.77 [0.64, 0.93]
Kim 2014 28 44 12 14 1.0% 0.74 [0.54, 1.01]
Kuroki 2021 480 762 129 173 0.0% 0.84 [0.76, 0.94]
Lin 2016 144 302 110 227 6.7% 0.98 [0.82, 1.18]
Mikus 2013 6 13 1 1 0.1% 0.62 [0.23, 1.65]
Monaco 2021 3 45 1 76 0.0% 5.07 [0.54, 47.26]
Nishi 2022 667 846 769 846 41.1% 0.87 [0.83, 0.90]
Papadopou-los 2015 57 79 201 281 4.7% 1.01 [0.86, 1.18]
Park 2014 21 41 30 55 1.4% 0.94 [0.64, 1.38]
Rastan 2010 284 383 105 134 8.3% 0.95 [0.85, 1.05]
Ro 2013 41 60 139 193 3.5% 0.95 [0.78, 1.15]
Sakamoto 2012 62 94 4 4 0.0% 0.73 [0.53, 1.01]
Shinn 2009 26 39 35 53 1.6% 1.01 [0.75, 1.35]
Smedira 2001 41 110 44 92 2.6% 0.78 [0.56, 1.08]
Tepper 2019 18 30 23 30 1.2% 0.78 [0.55, 1.11]
Unosawa 2012 2 8 16 39 0.0% 0.61 [0.17, 2.14]
Wang 2013 13 41 31 46 1.6% 0.47 [0.29, 0.77]
Xu 2016 7 11 4 5 0.3% 0.80 [0.43, 1.49]
Total (95% CI) 2910 3132 100.0% 0.90 [0.86, 0.93]
Total events 1847 2128
Heterogeneity: Chi² = 35.36, df = 30 (P = 0.23); I² = 15%
Test for overall effect: Z = 6.10 (P < 0.00001)
0.5 0.7 1 1.5 2
ECMO+IABP ECMO alone
C
ECMO+IABP ECMO alone Risk Ratio Risk Ratio
Study or Subgroup Events Total Events Total Weight M-H, Fixed, 95% CI M-H, Fixed, 95% CI
Acheampong 2016 6 14 5 10 0.3% 0.86 [0.36, 2.04]
Aoyama 2013 22 35 2 3 0.2% 0.94 [0.41, 2.18]
Arafat 2023 36 60 27 43 1.8% 0.96 [0.70, 1.30]
Asaumi 2005 3 6 1 8 0.0% 4.00 [0.54, 29.57]
Aso 2016 298 533 344 533 19.6% 0.87 [0.79, 0.96]
Aziz 2010 0 2 4 8 0.1% 0.33 [0.02, 4.55]
Barge Caballero 2019 18 52 25 77 1.2% 1.07 [0.65, 1.75]
Beurtheret 2013 17 27 38 60 1.3% 0.99 [0.70, 1.41]
Bjomsdottir 2022 73 114 66 114 3.8% 1.11 [0.90, 1.36]
Brechot 2017 28 63 35 63 2.0% 0.80 [0.56, 1.14]
Brink 2021 0 7 4 11 0.2% 0.17 [0.01, 2.69]
Char 2021 29 68 86 143 3.2% 0.71 [0.52, 0.96]
Chen 2005 1 6 2 4 0.1% 0.33 [0.04, 2.56]
Chen 2019 40 77 39 75 2.3% 1.00 [0.74, 1.36]
Chung 2011 7 14 3 6 0.2% 1.00 [0.38, 2.60]
Djordjevic 2021 92 129 35 43 3.0% 0.88 [0.73, 1.05]
Doll 2004 106 144 61 75 4.6% 0.91 [0.78, 1.05]
Elsharkawy 2010 15 22 134 211 1.4% 1.07 [0.79, 1.45]
Gass 2014 18 56 39 79 1.8% 0.65 [0.42, 1.01]
Hei 2011 5 11 20 57 0.4% 1.30 [0.62, 2.71]
Kida 2022 248 459 42 60 4.2% 0.77 [0.64, 0.93]
Kim 2014 28 44 12 14 1.0% 0.74 [0.54, 1.01]
Kuroki 2021 480 762 129 173 12.0% 0.84 [0.76, 0.94]
Lin 2016 144 302 110 227 7.2% 0.98 [0.82, 1.18]
Mikus 2013 6 13 1 1 0.1% 0.62 [0.23, 1.65]
Monaco 2021 3 45 1 76 0.0% 5.07 [0.54, 47.26]
Nishi 2022 667 846 769 846 0.0% 0.87 [0.83, 0.90]
Papadopou-los 2015 57 79 201 281 5.0% 1.01 [0.86, 1.18]
Park 2014 21 41 30 55 1.5% 0.94 [0.64, 1.38]
Rastan 2010 284 383 105 134 8.9% 0.95 [0.85, 1.05]
Ro 2013 41 60 139 193 3.8% 0.95 [0.78, 1.15]
Sakamoto 2012 62 94 4 4 0.5% 0.73 [0.53, 1.01]
Shinn 2009 26 39 35 53 1.7% 1.01 [0.75, 1.35]
Smedira 2001 41 110 44 92 2.7% 0.78 [0.56, 1.08]
Tepper 2019 18 30 23 30 1.3% 0.78 [0.55, 1.11]
Unosawa 2012 2 8 16 39 0.3% 0.61 [0.17, 2.14]
Wang 2013 13 41 31 46 1.7% 0.47 [0.29, 0.77]
Xu 2016 7 11 4 5 0.3% 0.80 [0.43, 1.49]
Total (95% CI) 3961 3106 100.0% 0.89 [0.85, 0.93]
Total events 2295 1897
Heterogeneity: Chi² = 41.19, df = 36 (P = 0.25); I² = 13%
Test for overall effect: Z = 5.48 (P < 0.00001)
0.5 0.7 1 1.5 2
ECMO+IABP ECMO alone

Supplementary Figure 5: Forest plot of in-hospital mortality in patients treated with venoarterial ECMO plus IABP and venoarterial ECMO. The updated studies in this meta-analysis from 13 June 2017 to 31 May 2023.

	ECMO+IABP		ECMO alone			Risk Ratio
Study or Subgroup	Events	Total	Events	Total	Weight	M-H, Fixed, 95% CI
Arafat 2023	36	60	27	43	2.3%	0.96 [0.70, 1.30]
Barge Caballero 2019	18	52	25	77	1.5%	1.07 [0.65, 1.75]
Bjornsdottir 2022	73	114	66	114	4.8%	1.11 [0.90, 1.36]
Brechot 2017	28	63	35	63	2.5%	0.80 [0.56, 1.14]
Brink 2021	0	7	4	11	0.3%	0.17 [0.01, 2.69]
Char 2021	29	68	86	143	4.0%	0.71 [0.52, 0.96]
Chen 2019	40	77	39	75	2.9%	1.00 [0.74, 1.36]
Djordjevic 2021	92	129	35	43	3.8%	0.88 [0.73, 1.05]
Kida 2022	248	459	42	60	5.4%	0.77 [0.64, 0.93]
Kuroki 2021	480	762	129	173	15.2%	0.84 [0.76, 0.94]
Monaco 2021	3	45	1	76	0.1%	5.07 [0.54, 47.26]
Nishi 2022	667	846	769	846	55.7%	0.87 [0.83, 0.90]
Tepper 2019	18	30	23	30	1.7%	0.78 [0.55, 1.11]
**Total (95% CI)**		**2712**		**1754**	**100.0%**	**0.87 [0.84, 0.90]**
Total events	1732		1281			

Heterogeneity: Chi² = 14.85, df = 12 (P = 0.25); I² = 19%

Test for overall effect: Z = 7.11 (P < 0.00001)

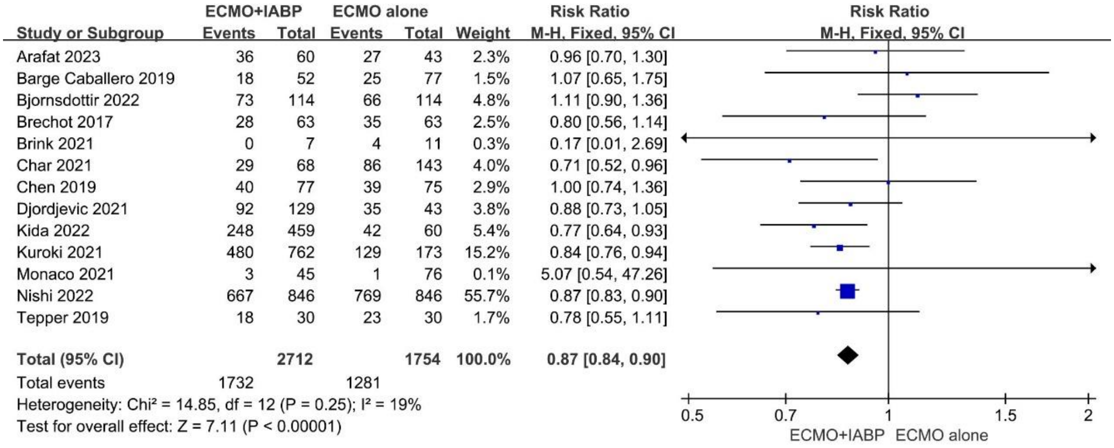

Supplementary Table 1: Characteristics of included studies before 2017

Study	Study type	Study period	Average years	Men, n(%)	Number of patients		Patient type	Peripheral ECMO, n(%)	Average time on ECMO	Survival to D/C,n(%)	Country/district
					ECMO	ECMO+IABP					
Acheampong 2016 [1]	RCS	01/2001-12/2013	41.0±39.3	14（58.3）	10	14	PCS	NA	8.4 ± 25.6 days	11 (45.8)	USA
Aoyama 2013 [2]	RCS	08/1993-08/2000	59.9 ± 13.5	35 (92.1)	3	35	IHD	38 (100)	126.5 ± 127.7 h	14 (36.8)	Japan
Asaumi 2005 [3]	RCS	01/1993-12/2001	38.4 ± 15.8	7 (50)	8	6	Myocarditis	14 (100)	130.0 ± 95.6 h	10 (71.4)	Japan
Aso 2016 [4]	RCS	07/2010-03/2013	65 ± 18.5	774 (72.6)	533	533	IHD, PCS	1066 (100)	2.4 days	424 (39.8)	Japan
Aziz 2010 [5]	RCS	06/2008-11/2008	45 ± 18.0	5 (50)	8	2	IHD, PCS	10 (100)	5.0 ± 4.4	6 (60)	USA
Beurtheret 2013 [6]	RCS	01/2005-12/2009	46 ± 15.0	59 (67.8)	60	27	IHD	87 (100)	9.0 ± 17.8 days	32 (36.8)	France
Chen 2005 [7]	RCS	01/1994-12/2001	37.4 ± 14.7	5 (50)	4	6	Myocarditis	8 (80)	118.7 ± 58.9 h	7（70）	Taiwan
Chen 2006 [8]	RCS	01/1994-12/2003	58 ± 10.0	33 (91.7)	5	31	IHD	36 (100)	108.5 ± 77.5 h	12 (33.3)	Taiwan
Chung 2011 [9]	RCS	05/2006-11/2009	67.7 ± 11.7	14 (70)	6	14	IHD	20 (100)	3.8 ± 4.3 days	10 (50)	Korea
Doll 2004 [10]	RCS	11/1997-07/2002	61.3 ± 12.1	160 (73.1)	75	144	PCS	54 (24.7)	2.8 ± 2.2 days	52 (23.7)	Germany
Elsharkawy 2010 [11]	RCS	01/1995-12/2005	57.5 ± 14.6	157 (67.4)	211	22	PCS	156 (67)	NA	84 (36.1)	USA

Gass 2014 [12]	RCS	01/2007-12/2012	57.3 ± 15.3	87 (64.4)	79	56	IHD, PCS	135 (100)	8.5 ± 7.1 days	78 (57.8)	USA
Guru 2015 [13]	RCS	05/2001-12/2014	56.0 ± 22.2	53 (52.5)	51	50	PCS	NA	100.0 ± 115.6 days	49 (48.5)	USA
Hei 2011 [14]	RCS	12/2004-12/2009	49.2 ± 13.3	52 (76.5)	57	11	PCS	67 (98.5)	114.6 ± 67.7 h	43 (63.2)	China
Kagawa 2012 [15]	RCS	01/2004-05/2011	63.0 ± 11.9	70 (81.4)	15	71	IHD	86 (100)	24.0 ± 42.2 h	25 (29.1)	Japan
Kim 2010 [16]	RCS	03/2010-07/2013	61.2 ± 11.3	48 (82.8)	14	44	IHD	NA	68.7 ± 17.4 h	18 (31.0)	Korea
Lin 2016 [17]	RCS	01/2002-12/2013	55.1 ± 14.0	399 (75.4)	227	302	IHD	529 (100)	4.0 ± 6.3 days	275 (52.0)	Taiwan
Mikus 2013	RCS	01/2007-08/2011	53.1 ± 14.3	9 (64.3)	1	13	PCS	6 (42.9)	9.0 ± 3.1 h	7 (50)	Italy
Papadopou-los 2015 [18]	RCS	12/2001-06/2013	62.0 ± 17.0	274 (76.1)	281	79	PCS	324 (90)	7.0 ± 1.0 days	108 (30)	Germany
Park 2014 [19]	RCS	01/2004-12/2011	64.9 ± 16.1	74 (77.1)	55	41	IHD	96 (100)	NA	45 (46.9)	Korea
Rastan 2010 [20]	RCS	05/1996-05/2008	63.5 ± 11.2	370 (71.6)	134	383	PCS	203 (39.3)	3.3 ± 2.9 days	128 (24.8)	Germany
Ro 2013 [21]	RCS	01/2005-08/2012	58.8 ± 15.3	154 (60.9)	193	60	PCS	244 (96.4)	71.0 ± 78.7 h	73 (28.9)	Korea
Sakamoto 2012 [22]	RCS	01/2000-12/2010	72.0 ± 12.0	65 (66.3)	4	94	IHD	98 (100)	68.9 ± 62.7 h	32 (32.7)	Japan
Shinn 2009 [23]	RCS	01/2006-12/2014	56.0 ± 18.0	59 (64.1)	53	39	IHD, PCS	92 (100)	90.9 ± 126.0 h	39 (42.4)	Korea

Smedira 2001 [24]	RCS	01/1992-06/1999	55.0 ± 18.0	145 (71.7)	92	110	PCS	153 (75.7)	NA	117 (57.9)	USA
Unosawa 2012 [25]	RCS	04/1992-06/2007	64.4 ± 12.5	35 (74.4)	39	8	PCS	32 (68.1)	63.5 ± 61.5 h	14 (29.8)	Japan
Wang 2013 [26]	RCS	01/2004-12/2011	65.0 ± 7.0	56 (64.4)	46	41	PCS	87 (100)	61.0 ± 37.0 h	43 (49.4)	China
Wu 2012 [27]	RCS	01/2003-06/2011	51.3 ± 13.9	40 (66.7)	16	44	IHD	60 (100)	97.3 ± 21.0 h	32 (53.3)	Taiwan
Xu 2016 [28]	RCS	01/2010-01/2016	62.3 ± 11.1	10 (62.5)	5	11	IHD	NA	119.3 ± 114.8 h	5 (31.3)	Australia

ECMO: extracorporeal membrane oxygenation; IABP: intra-aortic balloon pump; D/C: hospital discharge; RCS: retrospective cohort study; PCS: postcardiotomy cardiogenic shock; IHD: ischaemic heart disease; NA: not available

Supplementary Table 2: Newcastle-Ottawa Scale for Quality Assessment

Study	Selection				Comparability	Exposure			Scores
	Representativeness of the exposed cohort	Selection of the non exposed cohort	Ascertainment of exposure	Demonstration that outcome of interest was not present at start of study	Comparability of cohorts on the basis of the design or analysis	Assessment of outcome	Was follow-up long enough for outcomes to occur	Adequacy of follow up of cohorts	
Acheampong 2016	★	★	★	★	☆☆	★	★	★	7
Aoyama 2013	★	★	★	★	☆☆	★	★	★	7
Arafat 2023	★	★	★	★	☆☆	★	★	★	7
Asaumi 2005	★	★	★	★	☆☆	★	★	★	7
Aso 2016	★	★	★	★	★☆	★	★	★	8
Aziz 2010	★	★	☆	★	☆☆	★	★	★	6
Barge-Caballero 2019	★	★	★	★	☆☆	★	★	★	7
Beurtheret 2013	★	★	☆	★	☆☆	★	★	★	6
Bjornsdottir 2022	★	★	★	★	★☆	★	★	★	8
Brechot 2017	★	★	★	★	★☆	★	★	★	8
Brink 2021	★	★	★	★	☆☆	★	★	★	7
Char 2021	★	★	★	★	☆☆	★	☆	★	6
Chen 2005	★	★	★	★	☆☆	★	★	★	7
Chen 2006	★	★	★	★	☆☆	★	★	★	7
Chen 2019	★	★	★	★	☆☆	★	★	★	7
Chung 2011	★	★	★	★	☆☆	★	★	★	7
Djordjevic 2021	★	★	★	★	☆☆	★	★	★	7
Doll 2004	★	★	★	★	☆☆	★	★	★	7
Elsharkawy 2010	★	★	☆	★	☆☆	★	★	★	6
Gass 2014	★	★	★	★	★☆	★	★	★	8
Guru 2015	★	★	☆	★	★☆	☆	★	☆	5

Hei 2011	★	★	★	★	☆☆	★	★	★	7
Kagawa 2012	★	★	☆	★	☆☆	★	★	★	6
Kida 2022	★	★	★	★	☆☆	☆	★	☆	5
Kim 2014	★	★	☆	★	★☆	☆	★	☆	5
Kuroki 2021	★	★	★	★	☆★	★	★	★	8
Lin 2016	★	★	★	★	★☆	★	★	★	8
Mikus 2013	★	★	★	★	☆☆	★	★	★	7
Monaco 2021	★	★	★	★	☆☆	☆	★	★	6
Nishi 2022	★	★	★	★	☆★	★	★	★	8
Papadopou-los 2015	★	★	★	★	☆☆	★	★	★	7
Park 2014	★	★	★	★	★☆	★	★	★	8
Rastan 2010	★	★	★	★	☆☆	★	★	★	7
Ro 2013	★	★	★	★	★☆	★	★	★	8
Sakamoto 2012	★	★	★	★	☆☆	★	★	★	7
Shinn 2009	★	★	★	★	☆☆	★	★	★	7
Smedira 2001	★	★	★	★	☆☆	★	★	★	7
Tepper 2019	★	★	☆	★	☆☆	★	★	★	6
Unosawa 2012	★	★	☆	★	☆☆	★	★	★	6
Wang 2013	★	★	☆	★	☆☆	★	★	★	6
Wu 2012	★	★	☆	★	☆☆	★	★	★	6
Xu 2016	★	★	☆	★	☆☆	☆	★	☆	4

1. The VA-ECMO implantation method

The implantation techniques for VA-ECMO are categorized into central and peripheral approaches. Central ECMO implantation was performed by switching the inserted CPB cannulas from the right atrium and ascending aorta to the ECMO system or by direct re-cannulation. Cannulas

were fixed at the atrium, aorta and on the patient's body. Peripheral ECMO was surgically inserted with femoral-femoral cannulas.

2. The IABP implantation method

   In the recorded studies, IABP was inserted through a femoral sheath and with the tip located near the second rib with a 30 or 40 mL IABP balloon, which was judged according to the patient's parameters. The support was initiated at a 1:1 inflation–deflation to cardiac cycle ratio, triggering by the R wave of the electrocardiogram.

3. the ideal way of decannulation for VA ECMO and IABP

   ECMO weaning was initiated after haemodynamic stabilization. Concomitant diagnostics (echocardiography, laboratory parameters, chest X-ray) were used to evaluate weaning ability. Patients were assessed daily for possible VA-ECMO weaning using clinical and echocardiographic criteria as follows: baseline MBP >60 mmHg, receiving no or low-dose vasoactive agents and a pulsatile arterial waveform maintained for at least 24 h, $SvO_2 \geq 70\%$, hematocrit of 30–35%, absence of bleeding, tamponade or left heart distension, left ventricular ejection fraction (LVEF)≥35%, normal blood lactate levels and pulmonary blood oxygenation was not compromised. The VA-ECMO flow was decreased to 66% for 10-15 min, then to 33% and/or to a minimum of 1-1.5 L/min for another 10-15 min. Doppler echocardiography was repeated at each ECMO flow level. If the patient remained stable after prolonged complete circuit clamping, the machine was surgically removed and the mediastinum or femoral access surgically repaired. (Intensive Care Med (2011) 37:1738–1745; European Heart Journal: Acute Cardiovascular Care 2018, Vol. 7(1) 62–69).

   In patients with concomitant ECMO and IABP, IABP was left with ongoing 1:1 ECG-triggered counter pulsation for at least 24 hours after ECMO removal. IABP was removed after sequential reduction of IABP support.

The weaning criteria of IABP were the systolic blood pressure above 100 mmHg without inotropic agent after removal of ECMO. The support was decreased at a 1:3 inflation–deflation to cardiac cycle ratio when weaning program was initiated, and the patients were weaned off of IABP if the hemodynamic condition was stable.

The Doppler echocardiography parameters better separated weaned and non-weaned patients than any other parameters tested. So, the simple and easy-to-acquire Doppler echocardiography parameters as predictors of subsequent ECMO-weaning success in patients recovering from severe cardiogenic shock. (Intensive Care Med (2011) 37:1738–1745).

REFERENCES

1. Acheampong B, Johnson JN, Stulak JM, Dearani JA, Kushwaha SS, Daly RC, Haile DT, Schears GJ: **Postcardiotomy ECMO Support after High-risk Operations in Adult Congenital Heart Disease**. *Congenit Heart Dis* 2016, **11**(6):751-755.
2. Aoyama N, Imai H, Kurosawa T, Fukuda N, Moriguchi M, Nishinari M, Nishii M, Kono K, Soma K, Izumi T: **Therapeutic strategy using extracorporeal life support, including appropriate indication, management, limitation and timing of switch to ventricular assist device in patients with acute myocardial infarction**. *J Artif Organs* 2014, **17**(1):33-41.
3. Asaumi Y, Yasuda S, Morii I, Kakuchi H, Otsuka Y, Kawamura A, Sasako Y, Nakatani T, Nonogi H, Miyazaki S: **Favourable clinical outcome in patients with cardiogenic shock due to fulminant myocarditis supported by percutaneous extracorporeal membrane oxygenation**. *Eur Heart J* 2005, **26**(20):2185-2192.
4. Aso S, Matsui H, Fushimi K, Yasunaga H: **The Effect of Intraaortic Balloon Pumping Under Venoarterial Extracorporeal Membrane Oxygenation on Mortality of Cardiogenic Patients: An Analysis Using a Nationwide Inpatient Database**. *Crit Care Med* 2016, **44**(11):1974-1979.
5. Aziz TA, Singh G, Popjes E, Stephenson E, Mulvey S, Pae W, El-Banayosy A: **Initial experience with CentriMag extracorporal membrane oxygenation for support of critically ill patients with refractory cardiogenic shock**. *J Heart Lung Transplant* 2010, **29**(1):66-71.
6. Beurtheret S, Mordant P, Paoletti X, Marijon E, Celermajer DS, Léger P, Pavie A, Combes A, Leprince P: **Emergency circulatory support in refractory cardiogenic shock patients in remote institutions: a pilot study (the cardiac-RESCUE program)**. *Eur Heart J* 2013, **34**(2):112-120.
7. Chen YS, Yu HY, Huang SC, Chiu KM, Lin TY, Lai LP, Lin FY, Wang SS, Chu SH, Ko WJ: **Experience and result of extracorporeal membrane oxygenation in treating fulminant myocarditis with shock: what mechanical support should be considered first?** *J Heart Lung Transplant* 2005, **24**(1):81-87.

8. Chen JS, Ko WJ, Yu HY, Lai LP, Huang SC, Chi NH, Tsai CH, Wang SS, Lin FY, Chen YS: **Analysis of the outcome for patients experiencing myocardial infarction and cardiopulmonary resuscitation refractory to conventional therapies necessitating extracorporeal life support rescue**. *Crit Care Med* 2006, **34**(4):950-957.

9. Chung ES, Lim C, Lee HY, Choi JH, Lee JS, Park KH: **Results of Extracorporeal Membrane Oxygenation (ECMO) Support before Coronary Reperfusion in Cardiogenic Shock with Acute Myocardial Infarction**. *Korean J Thorac Cardiovasc Surg* 2011, **44**(4):273-278.

10. Doll N, Kiaii B, Borger M, Bucerius J, Krämer K, Schmitt DV, Walther T, Mohr FW: **Five-year results of 219 consecutive patients treated with extracorporeal membrane oxygenation for refractory postoperative cardiogenic shock**. *Ann Thorac Surg* 2004, **77**(1):151-157; discussion 157.

11. Elsharkawy HA, Li L, Esa WA, Sessler DI, Bashour CA: **Outcome in patients who require venoarterial extracorporeal membrane oxygenation support after cardiac surgery**. *J Cardiothorac Vasc Anesth* 2010, **24**(6):946-951.

12. Gass A, Palaniswamy C, Aronow WS, Kolte D, Khera S, Ahmad H, Cuomo LJ, Timmermans R, Cohen M, Tang GH *et al*: **Peripheral venoarterial extracorporeal membrane oxygenation in combination with intra-aortic balloon counterpulsation in patients with cardiovascular compromise**. *Cardiology* 2014, **129**(3):137-143.

13. Guru P, Sanghavi D, Seelhammer T, Schears G: **OUTCOME OF PATIENTS WITH REFRACTORY CARDIORESPIRATORY FAILURE MANAGED BY COMBINATION ECMO AND IABP**. *Critical Care Medicine* 2015, **43**(12 Suppl 1):45.

14. Hei F, Lou S, Li J, Yu K, Liu J, Feng Z, Zhao J, Hu S, Xu J, Chang Q *et al*: **Five-year results of 121 consecutive patients treated with extracorporeal membrane oxygenation at Fu Wai Hospital**. *Artif Organs* 2011, **35**(6):572-578.

15. Kagawa E, Dote K, Kato M, Sasaki S, Nakano Y, Kajikawa M, Higashi A, Itakura K, Sera A, Inoue I *et al*: **Should we emergently revascularize occluded coronaries for cardiac arrest?: rapid-response extracorporeal membrane oxygenation and intra-arrest percutaneous coronary intervention**. *Circulation* 2012, **126**(13):1605-1613.

16. Kim DK SG, Song PS, Chi NH, Yu HY, Chou NK: **Impact of concomitant use of intra-aortic balloon pump during percutaneous cardiopulmonary support in patients with cardiogenic shock complicating acute myocardial infarction**. *Eurointervention* 2010, **12**(S58).

17. Lin LY, Liao CW, Wang CH, Chi NH, Yu HY, Chou NK, Hwang JJ, Lin JL, Chiang FT, Chen YS: **Effects of Additional Intra-aortic Balloon Counter-Pulsation Therapy to Cardiogenic Shock Patients Supported by Extra-corporeal Membranous Oxygenation**. *Sci Rep* 2016, **6**:23838.

18. Papadopoulos N, Marinos S, El-Sayed Ahmad A, Keller H, Meybohm P, Zacharowski K, Moritz A, Zierer A: **Risk factors associated with adverse outcome following extracorporeal life support: analysis from 360 consecutive patients**. *Perfusion* 2015, **30**(4):284-290.

19. Park TK, Yang JH, Choi SH, Song YB, Hahn JY, Choi JH, Sung K, Lee YT, Gwon HC: **Clinical impact of intra-aortic balloon pump during extracorporeal life support in patients with acute myocardial infarction complicated by cardiogenic shock**. *BMC Anesthesiol* 2014, **14**:27.

20. Rastan AJ, Dege A, Mohr M, Doll N, Falk V, Walther T, Mohr FW: **Early and late outcomes of 517 consecutive adult patients treated with extracorporeal membrane oxygenation for refractory postcardiotomy cardiogenic shock**. *J Thorac Cardiovasc Surg* 2010, **139**(2):302-311, 311.e301.
21. Ro SK, Kim JB, Jung SH, Choo SJ, Chung CH, Lee JW: **Extracorporeal life support for cardiogenic shock: influence of concomitant intra-aortic balloon counterpulsation**. *Eur J Cardiothorac Surg* 2014, **46**(2):186-192; discussion 192.
22. Sakamoto S, Taniguchi N, Nakajima S, Takahashi A: **Extracorporeal life support for cardiogenic shock or cardiac arrest due to acute coronary syndrome**. *Ann Thorac Surg* 2012, **94**(1):1-7.
23. Shinn SH, Lee YT, Sung K, Min S, Kim WS, Park PW, Ha YK: **Efficacy of emergent percutaneous cardiopulmonary support in cardiac or respiratory failure: fight or flight?** *Interact Cardiovasc Thorac Surg* 2009, **9**(2):269-273.
24. Smedira NG, Moazami N, Golding CM, McCarthy PM, Apperson-Hansen C, Blackstone EH, Cosgrove DM, 3rd: **Clinical experience with 202 adults receiving extracorporeal membrane oxygenation for cardiac failure: survival at five years**. *J Thorac Cardiovasc Surg* 2001, **122**(1):92-102.
25. Unosawa S, Sezai A, Hata M, Nakata K, Yoshitake I, Wakui S, Kimura H, Takahashi K, Hata H, Shiono M: **Long-term outcomes of patients undergoing extracorporeal membrane oxygenation for refractory postcardiotomy cardiogenic shock**. *Surg Today* 2013, **43**(3):264-270.
26. Wang JG, Han J, Jia YX, Zeng W, Hou XT, Meng X: **Outcome of veno-arterial extracorporeal membrane oxygenation for patients undergoing valvular surgery**. *PLoS One* 2013, **8**(5):e63924.
27. Wu MY, Lee MY, Lin CC, Chang YS, Tsai FC, Lin PJ: **Resuscitation of non-postcardiotomy cardiogenic shock or cardiac arrest with extracorporeal life support: the role of bridging to intervention**. *Resuscitation* 2012, **83**(8):976-981.
28. Xu J, Leung D, Rajaratnam R, Mussap C, French J, Juergens C, Parr M, Lo S: **Contemporary Practice of Veno-Arterial Extracorporeal Membrane Oxygenation (VA-ECMO) at a Single Non-Transplant Centre: Survival Prediction Scores, Indications and Outcomes**. *Heart, Lung and Circulation* 2016, **25**:S171.
